# The application value of two-dimensional speckle tracking imaging technology in evaluating the functions of the left atrium and left atrial appendage in patients with non-valvular atrial fibrillation: A comparative cross-sectional study

**DOI:** 10.1097/MD.0000000000051377

**Published:** 2026-11-06

**Authors:** Xianghong Guan, Qiankun Chang, Limin Lu, Wenying Wu, Liang Fang, Chunqiang Bai, Peiming Feng

**Affiliations:** a Ultrasound Department of Southern Campus, Affiliated Hospital of Chengde Medical University, Chengde, Hebei, China.

**Keywords:** atrial fibrillation, echocardiography, left atrial appendage, speckle tracking imaging, strain

## Abstract

Atrial fibrillation (AF) is associated with increased risk of stroke due to left atrial appendage (LAA) thrombus formation. Two-dimensional speckle tracking imaging (2D-STI) may provide valuable insights into left atrial and LAA functional changes associated with thrombus formation. This study aimed to evaluate the functions of the left atrium (LA) and LAA in patients with non-valvular AF by using 2D-STI and explore echocardiographic parameters associated with the presence of LAA thrombus. In this comparative cross-sectional study, consecutive patients with non-valvular AF who were scheduled for radiofrequency ablation were screened. Finally, 68 eligible AF patients and 30 age-matched healthy controls were included. All AF patients underwent preoperative transthoracic echocardiography, transesophageal echocardiography, and two-dimensional speckle tracking imaging, and were classified according to the presence or absence of LAA thrombus detected by transesophageal echocardiography into the thrombus group (n = 32) and the non-thrombus group (n = 36). The healthy controls were included as a reference group for comparison of echocardiographic parameters. The left atrial appendage emptying velocity (LAAEV), left atrial global longitudinal strain (LAGLS), and LAA global longitudinal strain (LAAGLS) were measured and compared between groups. Compared with the control group, AF patients showed significantly reduced LAAEV, LAGLS, and left atrial appendage global longitudinal strain (all *P* < .05). In AF patients, the thrombus group had significantly lower LAAEV, LAGLS, and left atrial appendage global longitudinal strain compared to the non-thrombus group (all *P* < .05). Multivariable logistic regression analysis showed that LAAEV and left atrial strain during reservoir phase were significantly associated with the presence of LAA thrombus. 2D-STI can quantitatively evaluate the functional changes of the LA and LAA. LAAEV and left atrial strain during reservoir phase may serve as useful echocardiographic markers for assisting clinical evaluation of LAA thrombus.

## 1. Introduction

Atrial fibrillation (AF) is one of the common arrhythmias in clinical practice. Patients lose the regular and orderly electrical activity of the atria and present rapid and disordered fibrillation waves. It is a serious disease with disordered electrical activity of the atria.^[1]^ The incidence rate among adults is 2%−3%. One of the main hazards of non-valvular AF is stroke or thromboembolic events. Approximately 15% −20% of unexplained ischemic strokes are caused by cardiogenic factors, and more than half of the occurrence of cardiogenic strokes is related to non-valvular AF.^[2]^ Importantly, compared with non-AF strokes, AF-related strokes have an increased mortality rate and a poorer prognosis.^[3]^ Since the LAA is the predominant site of thrombus formation, more than 90% of thrombi in patients with non-valvular AF originate from this area, although thrombi may occasionally occur in the LA body. Radiofrequency ablation is an important means for treating AF. However, before the operation, it is necessary to clarify whether there is thrombus formation in the left atrium (LA) to prevent the occurrence of operation-related thromboembolic events.^[4]^ Therefore, early identification and clinical evaluation of LAA thrombus in patients with non-valvular AF are of great significance for guiding clinical treatment and improving prognosis.

Transesophageal Echocardiography (TEE) has the characteristics of high repeatability and high diagnostic accuracy. It can evaluate the structural function of the LAA in AF and observe whether there is a thrombus before the operation, which plays an important role in improving the safety of treatment and prognosis.^[5]^ Two-dimensional speckle tracking imaging (2D-STI) is not affected by cardiac motion and the contraction of adjacent segments. It can detect changes in compliance before the LA undergoes deformation. Transesophageal ultrasound can quantitatively evaluate the functional changes of the LA and LAA in real time and can observe whether there is thrombus formation in the LAA. This study applied 2D-STI and TEE to measure functional changes of the LA and LAA in patients with non-valvular AF and explored their clinical value in identifying echocardiographic parameters associated with LAA thrombus.

## 2. Materials and methods

### 2.1. Research subjects

This comparative cross-sectional observational study was conducted at the Affiliated Hospital of Chengde Medical University from December 2019 to June 2023. During the study period, consecutive patients with non-valvular AF who were scheduled to undergo radiofrequency ablation were screened for eligibility. No prespecified number or ratio of thrombus and non-thrombus patients was intentionally recruited. Of these, 90 patients were excluded due to: incomplete echocardiographic data (n = 31), poor image quality precluding adequate strain analysis (n = 24), previous cardiac surgery (n = 16), significant valvular heart disease (n = 12), and cardiomyopathy (n = 7). Finally, 68 patients were included in the final analysis. All patients underwent transthoracic echocardiography (TTE) and TEE before surgery. All eligible AF patients underwent TTE, TEE, and two-dimensional speckle tracking imaging before radiofrequency ablation. During TEE examination, both the LA body and the LAA were systematically evaluated for thrombus. Because the primary outcome of this study was LAA thrombus, AF patients were classified according to the presence or absence of LAA thrombus identified by TEE into the thrombus group with 32 cases (20 males and 12 females, aged 41 to 78 years, mean age 61.75 ± 8.85 years) and the non-thrombus group with 36 cases (28 males and 8 females, aged 34–73 years, mean age 58.61 ± 9.77 years). Additionally, 30 age-matched healthy individuals were recruited separately as a reference group for comparison of echocardiographic parameters, rather than as controls for the thrombus prediction analysis (Fig. 1).

**Figure 1.**
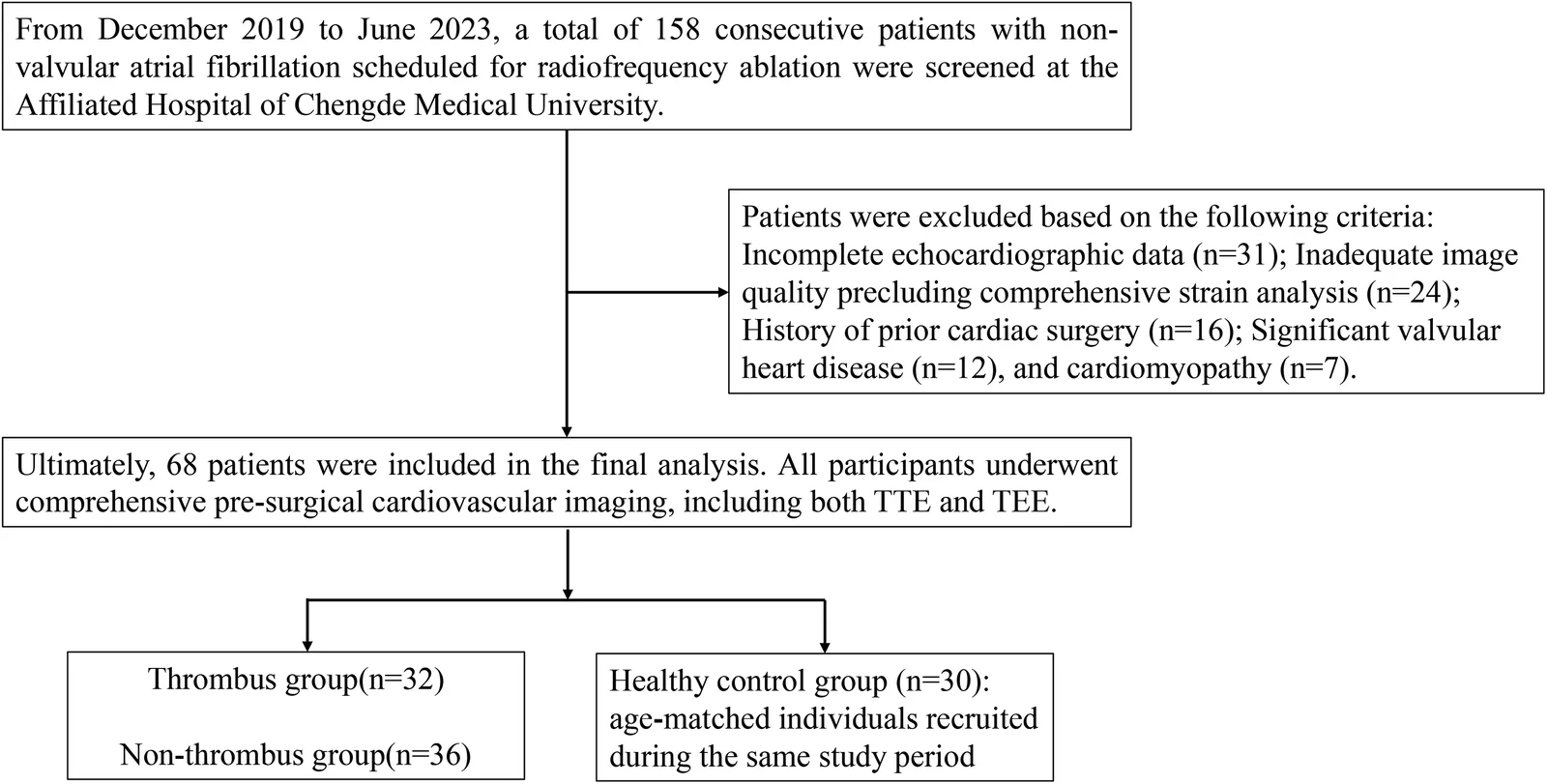
Patient selection flow diagram. TEE = Transesophageal echocardiography, TTE = Transthoracic echocardiography.

Inclusion criteria: Patients diagnosed with non-valvular AF by electrocardiogram, with a disease course of more than 6 months; All underwent transesophageal and TTE examinations, and the image quality was excellent.Exclusion criteria: Rheumatic valvular heart disease, congenital heart disease, cardiomyopathy and old myocardial infarction; A history of previous cardiac surgery; Incomplete data and poor image quality. Thirty individuals without arrhythmia who underwent screening for patent foramen ovale during the same period were selected as the healthy reference group, including 20 males and 10 females, aged 37 to 78 years, with a mean age of 60.03 ± 9.71 years. This group was used only to provide reference values for LA and LAA functional parameters and was not included in the analysis of factors associated with LAA thrombus among AF patients. There was no statistically significant difference in gender and age among each group (*P* > .05).

### 2.2. Ethical considerations

This study was approved by the Medical Ethics Committee of the Affiliated Hospital of Chengde Medical University (Approval No.: CYFYLL2020263). All procedures performed in this study were in accordance with the ethical standards of the institutional research committee and with the 1964 Helsinki Declaration and its later amendments. Written informed consent was obtained from all patients and their families prior to participation in the study.

### 2.3. Instruments and methods

#### 2.3.1. Blinding and bias-reduction procedures

Complete procedural blinding was not feasible during TEE because left atrial appendage (LAAEV) measurement and LAA thrombus assessment were performed during the same examination session. To reduce potential observer bias, LAAEV measurements were obtained and recorded before systematic evaluation for thrombus presence. All stored echocardiographic images for strain analysis were analyzed offline by 2 experienced sonographers, each with more than 3 years of speckle tracking experience, who were blinded to patients’ thrombus status and clinical information, including CHA_2_DS_2_-VASc score, medical history, and laboratory results. In addition, the presence of LAA thrombus was determined by consensus of 2 independent observers who were not involved in the offline strain analysis.

#### 2.3.2. Echocardiography examination

For TTE examination, the Philips EPIQ 7C color Doppler ultrasound diagnostic apparatus was used, with an S5–1 probe and a frequency ranging from 1 to 5 MHz. The patient was placed in the left lateral decubitus position. The anteroposterior diameter of the left atrium (LAD) was measured, and the apical 2-chamber and 4-chamber views with the LA as the main focus were obtained. The maximum volume of the left atrium (left atrial maximum volume [LAVmax]), the minimum volume of the left atrium (left atrial minimum volume [LAVmin]), and the left atrial emptying fraction (LAEF) were measured by the biplane Simpson method. Six complete cardiac cycle dynamic images were collected and processed offline. For TEE examination, the Philips EPIQ 7C color Doppler ultrasound diagnostic apparatus was also used, with an S7–2t probe and a frequency ranging from 3.0 to 7.0 MHz. The structure of the LAA was clearly displayed at the mid-esophageal level. Taking the line connecting the entrance of the left superior pulmonary vein and the mitral annulus as the boundary, the inner diameter of the left atrial appendage opening was measured. The distance from the midpoint of the LAA opening to the top of the LAA was measured to obtain the depth of the left atrial appendage height (LAAH). The peak blood flow emptying velocity of the LAAEV was measured by pulsed Doppler. Six complete cardiac cycle dynamic images were collected and processed offline. All parameters were measured 3 times repeatedly and the average values were taken (Fig. 2).

**Figure 2.**
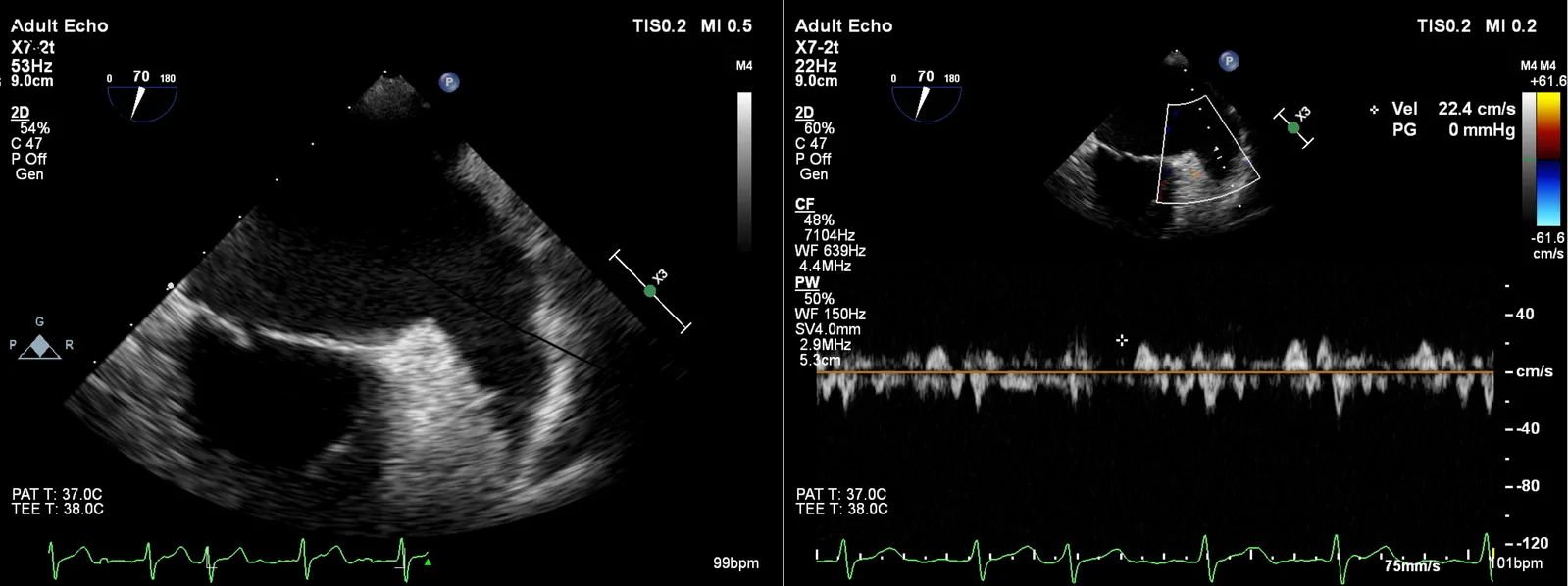
Measurement of 2-dimensional and spectral Doppler parameters of transesophageal echocardiography.

#### 2.3.3. Image processing and data analysis

The Philips QLAB 13.0 software was used for strain analysis of the LA and LAA. For TTE, the LA strain mode was applied. With the end-diastolic phase of the electrocardiogram as the zero point, the peak strain in the left atrial reservoir phase (LASr), the peak strain in the left atrial conduit phase (LAScd), and the peak strain in the left atrial strain during contraction phase (LASct) were measured. For TEE, the Philips QLAB 13.0 software was used to measure the global longitudinal strain of the left atrial appendage (LAAGLS) and the left atrial appendage ejection fraction (LAAEF). The above operations were completed by sonographers with 3 years of experience in speckle tracking analysis (Figs. 3 and 4).

**Figure 3.**
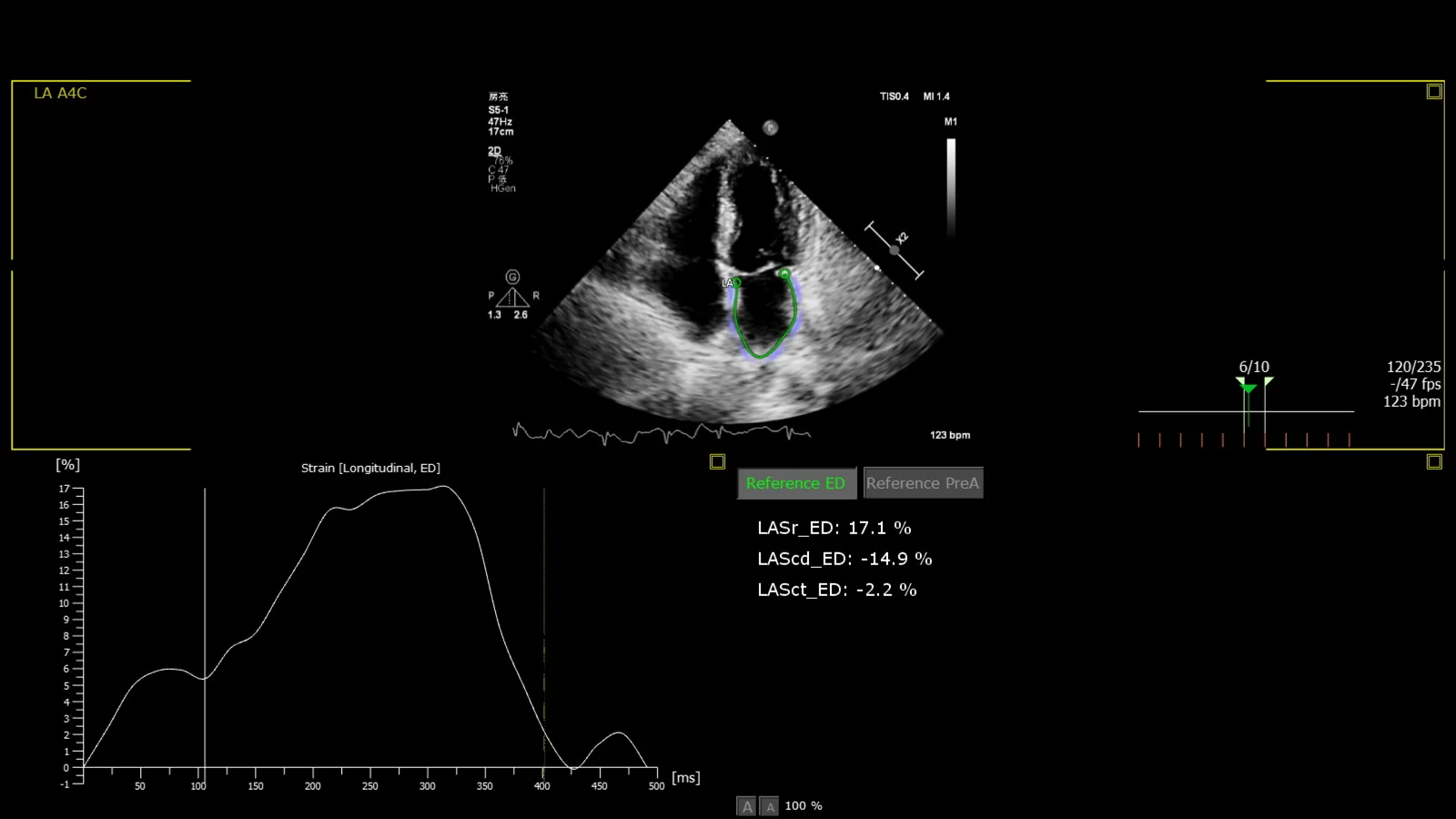
Left atrial strain analysis in patients with atrial fibrillation.

**Figure 4.**
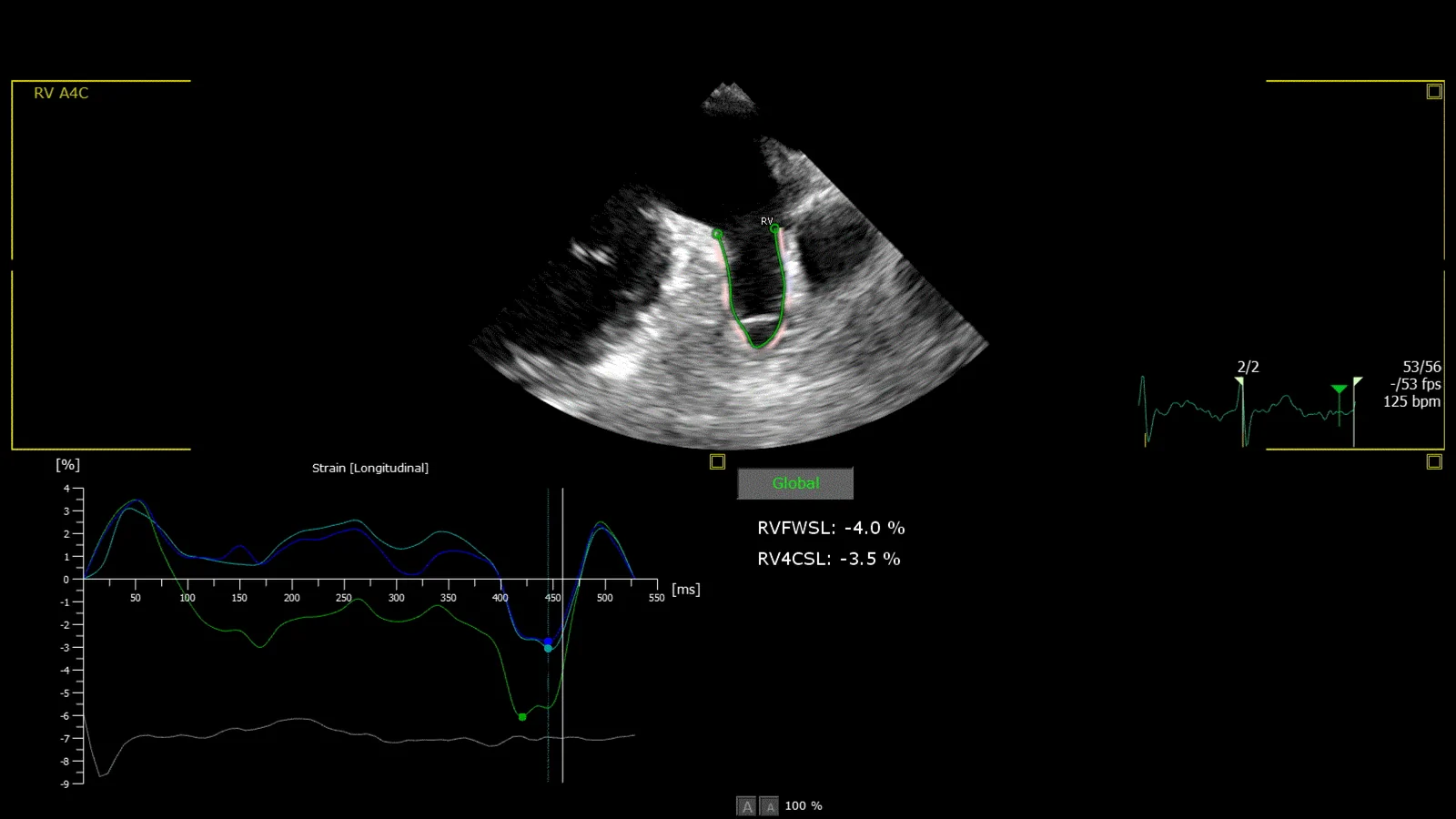
Analysis of the global strain of the left atrial appendage using the RV Strain mode.

3.After the patients were admitted to the hospital, the CHA2DS2-VASc score was used to assess the risk of AF-related thrombosis. The scoring criteria were as follows: Heart failure or left ventricular ejection fraction (LVEF) <40% was scored as 1 point; concurrent hypertension was scored as 1 point; age >75 years was scored as 2 points; age between 65 and 74 years was scored as 1 point; concurrent diabetes mellitus was scored as 1 point; concurrent stroke or thrombosis formation was scored as 2 points; concurrent vascular diseases was scored as 1 point; being female was scored as 1 point.

### 2.4. Statistical processing

Statistical analysis was performed using SPSS 26.0 software. Measurement data were expressed as mean±standard deviation (x±s). One-way analysis of variance was used for comparison of various parameters. Spaerman correlation analysis was used to analyze the correlation between the CHA2DS2-VASc score and various parameters. Among patients with non-valvular AF, multivariable logistic regression analysis was performed using the presence or absence of LAA thrombus as the dependent variable, with adjustment for CHA_2_DS_2_-VASc score, left atrial diameter (LAD), and LVEF to identify factors associated with LAA thrombus. The healthy reference group was not included in this regression model. ROC curves of the LA and LAA were drawn to evaluate the discriminatory ability of these parameters for distinguishing patients with and without LAA thrombus. A *P* value <.05 indicates a statistically significant difference.

## 3. Results

### 3.1. Comparison of left atrial function among 3 groups of patients

One-way analysis of variance was used to compare the differences in LAD, LAVmax, LAVmin, LAEF, LASr, LAScd, and LASc among the 3 groups of samples, and the results are shown in Table 1. Compared with the control group, there were statistically significant differences in LAD, LAVmax, LAVmin, LAEF, LASr, LAScd, and LASct between the non-thrombosis group and the thrombosis group (*P *< .05). LAD, LAVmax, and LAVmin showed an increasing trend, while LAEF, LASr, LAScd, and LASct showed a decreasing trend. When comparing the thrombosis group with the non-thrombosis group, there were statistically significant differences in LAD, LAVmax, LAEF, LASr, and LASct (*P* < .05), as shown in Table 1.

**Table 1:** Comparison of Left Atrial Function among 3 Groups of Patients (x ± s).

Group	n	LAD	LAVmax	LAVmin	LAEF	LASr	LAScd	LASct
Thrombosis group	32	39.25 ± 3.86*^,^†	60.06 ± 9.32*^,^†	38.16 ± 10.42*	43.94 ± 5.61*^,^†	24.03 ± 3.71*^,^†	−15.53 ± 3.5*	−7.84 ± 2.95*#
Non-thrombosisGroup	36	36.22 ± 3.14	51.06 ± 10.01	41.86 ± 8.94	51.81 ± 7.29	28.97 ± 4.59	−16.83 ± 3.95	−13.17 ± 3.56
Control Group	30	32.97 ± 3.34	39.97 ± 5.99	16.7 ± 5.01	66.03 ± 3.21	42.17 ± 4.28	−25.87 ± 4.13	−16.4 ± 3.57
*F*		32.936	53.36	75.352	153.209	200.352	80.08	77.95
*P*		<.001	<.001	<.001	<.001	<.001	<0.001	<0.001

LAD = left atrial diameter, LAEF = left atrial emptying fraction, LAScd = left atrial strain during conduit phase, LASct = left atrial strain during contraction phase, LASr = left atrial strain during reservoir phase, LAVmax = left atrial maximum volume, LAVmin = left atrial minimum volume.

* Indicates comparison with the normal group.

† Indicates comparison with the non-thrombosis group.

### 3.2. *Comparison of* LAA *function among 3 groups of patients*

There was no statistically significant difference in the comparison of left atrial appendage opening and LAAH among the 3 groups of patients (*P* > .05). Compared with the control group, there were statistically significant differences in LAAEV, LAAEF, and left atrial appendage global longitudinal strain (LAAGLS) between the thrombosis group and the non-thrombosis group (*P* < .05), as shown in Table 2.

**Table 2:** Comparison of left atrial appendage function among 3 groups of patients (x ± s).

Group	n	LAAD	LAAH	LAAEV	LAAEF	LAAGLS
Thrombosis group	32	14.09 ± 1.41	26.19 ± 4.77	45.38 ± 4*^,^†	27.53 ± 5.71*^,^†	9.84 ± 2.12*^,^†
Non-thrombosis group	36	14.06 ± 1.24	28.11 ± 4.62	56.06 ± 4.07	29.72 ± 6.44	12.86 ± 2.27
Control group	30	14.07 ± 1.26	26.1 ± 4.15	59.2 ± 4.69	39.73 ± 5.14	17.6 ± 2.97
F		0.011	2.353	140.562	46.316	109.055
*P*		.989	.099	<.001	<.001	<.001

LAAD = left atrial appendage depth, LAAEF = left atrial appendage ejection fraction, LAAEV = left atrial appendage emptying velocity, LAAGLS = left atrial appendage global longitudinal strain, LAAH = left atrial appendage height.

* Indicates comparison with the normal group.

† Indicates comparison with the non-thrombosis group.

### 3.3. Correlation between measured parameters of 3 groups of patients and the CHA2DS2-VASc score

The CHA2DS2-VASc scores of the thrombosis group, non-thrombosis group and control group were 4.09 ± 1.05, 1.64 ± 1.07 and 1.80 ± 1.67 points respectively, and the difference was statistically significant (*F* = 61.561, *P* < .001); there was no statistically significant difference between the non-thrombosis group and the control group (*P* > .05). The parameters of the LA and LAA of patients with AF that were significantly correlated with the CHA2DS2-VASc score were LAD (*R* = 0.342, *P* < .001), LAVmax (*R* = 0.337, *P *< .001), LAEF (*r* = −0.454, *P* < .001), LASr (*r* = −0.467, *P* < .001), LASct (*R* = 0.587, *P* < .001), LAAH (*r* = −0.216, *P* = .013), LAAEV (*r* = −0.616, *P* < .001), LAAEF (*r* = −0.279, *P* = .001) and LAAGLS (*r* = −0.559, *P* < .001), among which the correlation between LAAEV and the CHA2DS2-VASc score was the best. (Fig. 5)

**Figure 5.**
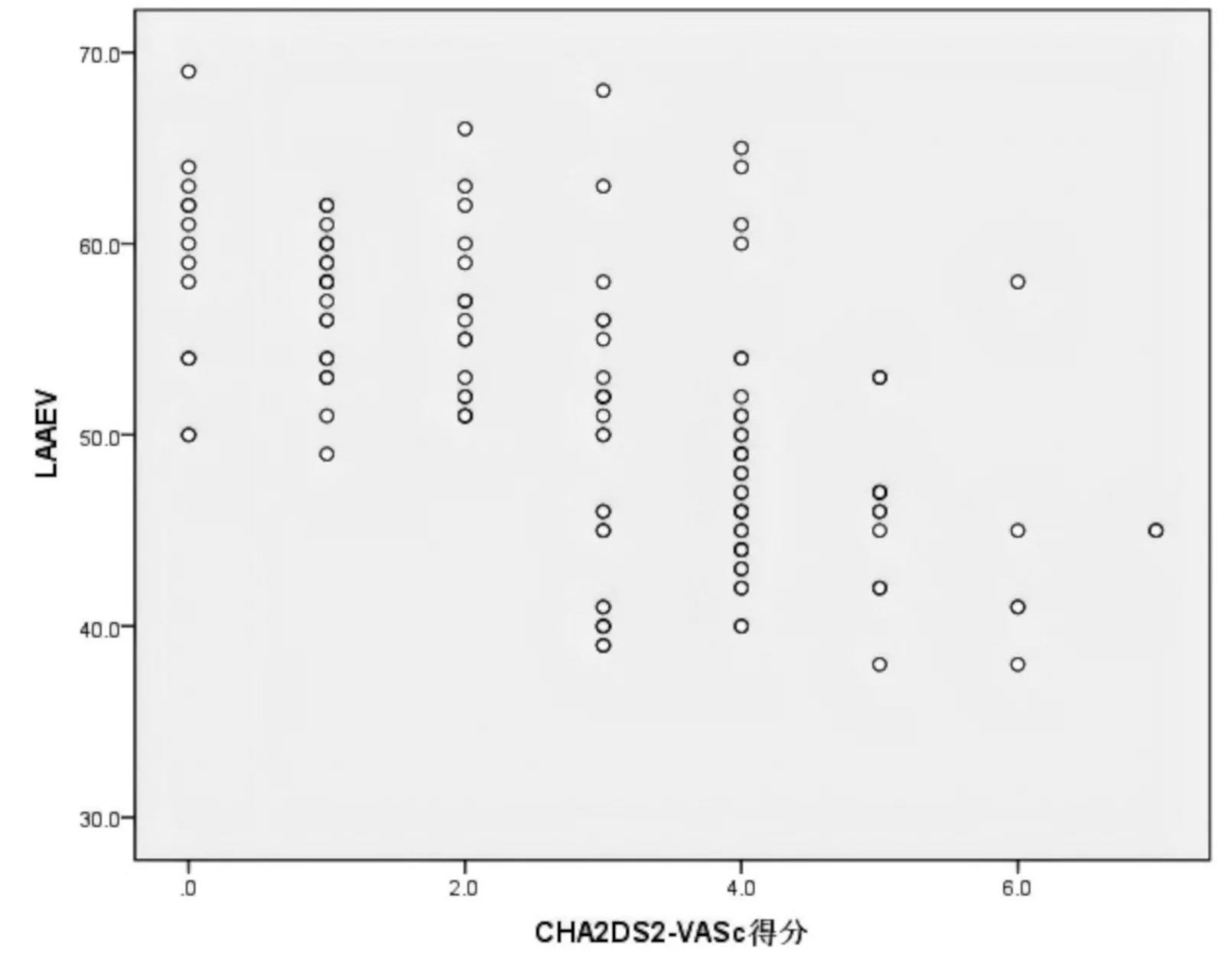
Scatter plot of LAAEV and CHA2DS2-VASc scores. LAAEV = left atrial appendage emptying velocity.

### 3.4. Logistic regression analysis of factors associated with LAA thrombus among patients with non-valvular AF

Multivariable logistic regression analysis was performed adjusting for CHA_2_DS_2_-VASc score, LAD, and LVEF. After adjustment for these clinically relevant covariates, LAAEV (adjusted odds ratio [aOR] = 0.88, 95% confidence interval (CI): 0.81–0.96, *P* = .004) and LASr (aOR = 0.86, 95%CI: 0.78–0.95, *P* = .003) were significantly associated with the presence of LAA thrombus. LAD showed a positive but nonsignificant association with LAA thrombus (aOR = 1.08, 95%CI: 0.98–1.19, *P* = .114), while CHA_2_DS_2_-VASc score was strongly associated with LAA thrombus (aOR = 2.10, 95%CI: 1.43–3.09, *P* < .001). The results are shown in Table 3.

**Table 3:** Multivariable logistic regression analysis of factors associated with LAA thrombus.

Variable	B	S.E.	Wald	*d*f	*P*	OR	95% CI
LAAEV	−0.128	0.045	8.132	1	.004	0.88	0.81–0.96
LASr	−0.156	0.052	8.973	1	.003	0.86	0.78–0.95
LAD	0.076	0.048	2.501	1	.114	1.08	0.98–1.19
CHA_2_DS_2_-VASc	0.742	0.196	14.336	1	<.001	2.10	1.43–3.09
LVEF	-0.038	0.027	1.975	1	.160	0.96	0.91–1.02

Multivariate logistic regression analysis was performed using the backward stepwise method; Variables with *P* < .05 were considered statistically significant factors associated with LAA thrombus; OR values <1 indicate protective factors; OR values >1 indicate risk factors.

B = regression coefficient, CI = confidence interval, df = degrees of freedom, LAAEV = left atrial appendage emptying velocity, LAD = left atrial diameter, LASr = left atrial strain rate, LVEF = left ventricular ejection fraction, OR = odds ratio, S.E. = Standard error, CHA_2_DS_2_-VASc score.

### 3.5. Diagnostic value of each parameter for LAA thrombus

The discriminatory value of parameters that were significant in the logistic regression was evaluated using receiver operating characteristic (ROC) analysis. The results showed that the areas under the curve of LASr, LAAEV, and LAD were 0.974, 0.793, and 0.714 respectively, as shown in Figure 2. The sensitivity of LASr was 72.2% and the specificity was 71.9%. The sensitivity of LAAEV was 86.1% and the specificity was 93.7%. The sensitivity of LAD was 91.7% and the specificity was 37.5%. These results indicate that LAAEV showed the highest discriminatory value for distinguishing AF patients with and without LAA thrombus, with a Youden index of 0.798 and a cutoff value of 51.5 cm/s. In this study population, LAAEV <51.5 cm/s was associated with the presence of LAA thrombus (Fig. 6).

**Figure 6.**
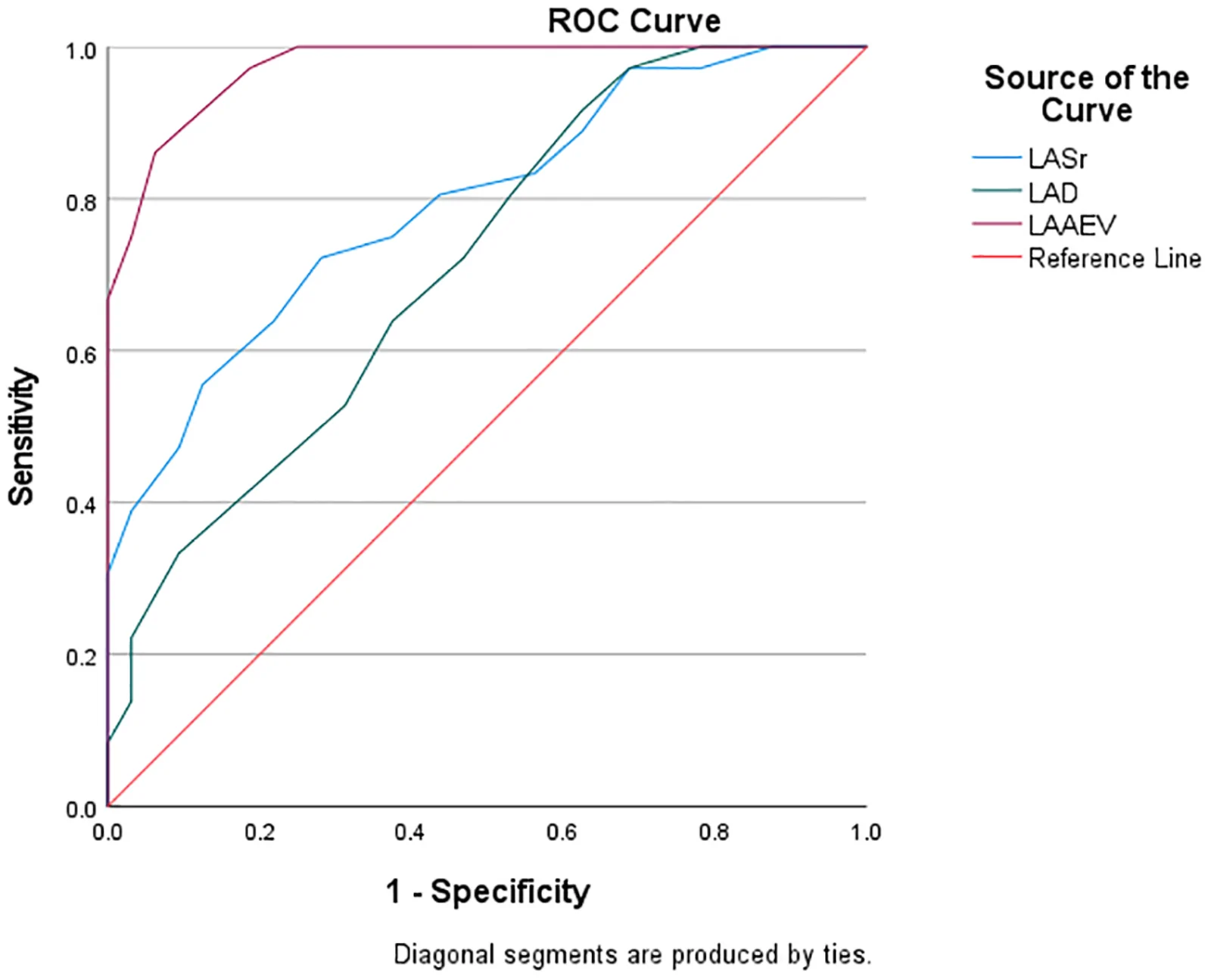
Receiver operating characteristic (ROC) curves for discriminating the presence of left atrial appendage thrombus in patients with non-valvular atrial fibrillation. ROC curves comparing the discriminatory performance of 3 echocardiographic parameters: LASr (Left Atrial Strain during Reservoir Phase, blue line), LAD (Left Atrial Diameter, green line), and LAAEV (Left Atrial Appendage Emptying Velocity, purple line) for distinguishing patients with and without left atrial appendage thrombus. The red diagonal line represents the reference line (AUC = 0.5, no discrimination). The closer the curve to the upper left corner, the better the diagnostic performance. AUC = area under the curve.

## 4. Discussion

The LA is an important hub connecting the left ventricle and the pulmonary veins, and it has 3 functions: Storage function: which is related to the volume of the left ventricle; Conduit function: which is related to the relaxation and compliance of the left ventricle; Pump function: which is related to venous return (preload) and left ventricular end-diastolic pressure (postload).^[6]^ Therefore, changes in the structure and function of the LA reflect diseases related to preload and postload. During AF, the normal contraction rhythm of the LA disappears, and the turbulent blood damages the myocardium to varying degrees, resulting in mechanical and electrical remodeling of the LA, myocardial fibrosis in the LA, and increased stiffness of the LA. Evaluating left atrial function through deformation strain and strain rate is a simple, accurate, and reproducible method, and it is more sensitive than traditional echocardiography. It can even show changes in left atrial function before changes in left ventricular volume.^[7]^In this study, two-dimensional speckle tracking echocardiography (2D-STE) was applied to conduct preoperative structural and functional assessments of the LA and left atrial appendage (LAA) in patients with AF who needed radiofrequency ablation in the Affiliated Hospital of Chengde Medical University from December 2019 to June 2023. Strain parameters were obtained, and then a comprehensive assessment was carried out by combining the conventional two-dimensional data of the LAA and LA. It was found that the compliance of the LA in patients with AF decreased, and the compliance in the group with thrombus was even lower than that in the group without thrombus. Meanwhile, through TEE assessment, LAAEV showed the strongest correlation with the CHA2DS2-VASc score and the highest discriminatory value for the presence of LAA thrombus. LASr and LAD also showed discriminatory value for distinguishing AF patients with and without LAA thrombus.

The research by Liu Jia et al^[8]^ showed that left atrial enlargement is related to the severity of AF and left atrial remodeling. In addition, left ventricular compliance, left ventricular filling pressure, the left atrial myocardial wall, and the pressure difference between the atrium and the ventricle are all closely related to left atrial enlargement. Left atrial enlargement reflects that the left ventricle is in a state of long-term elevation of filling pressure. Therefore, left atrial dysfunction is a powerful indicator of severe impairment of left ventricular diastolic function. The maximum volume of the LA can serve as an independent factor for adverse cardiovascular prognosis. TTE can measure the LAD and use the biplane Simpson method to measure the maximum volume of the left atrium (LAVmax), the minimum volume of the left atrium (LAVmin), and the LAEF. Wang Yanan et al^[9]^ found in their research that the increase in LAVmin in patients with AF can reflect atrial enlargement and impairment of systolic function, and the gradual decrease in LAEF is consistent with the progressive aggravation of atrial remodeling in AF. In this study, it was found that compared with the control group, the LAD, LAVmax, and LAVmin in the thrombus group and the non-thrombus group showed an increasing trend, while the LAEF showed a decreasing trend, which is consistent with previous studies.

Two-dimensional speckle tracking echocardiography (2D-STE) is a new method for noninvasive assessment of the functions of the LA and LAA. Using this technique, abnormal atrial myocardial movements can be detected early without angular dependence. Compared with previous techniques such as tissue Doppler, 2D-STE can noninvasively, rapidly, and quantitatively evaluate the local and overall deformation and function of the myocardium. In the past, 2D-STE was often used to assess left ventricular function. In recent years, its application in the assessment of left atrial (LA) function has received great attention. It uses strain to represent the deformation of the myocardium during contraction or relaxation^[10]^ and can detect subclinical myocardial injuries that cannot be detected by volume parameters earlier. According to the new EACVI/EHRA expert consensus, the LA strain parameters obtained by 2D-STE are promising indicators in the assessment of patients with AF.^[11]^ As early as 2013, Cao Sheng et al^[12]^ used the 2D-STI technique to assess the left atrial function of patients with AF. The results showed that when AF occurred, the reservoir function, conduit function, and pump function of the LA all significantly decreased, which is consistent with the results of this study. Moreover, this study found that the left atrial function in the thrombus group was lower than that in the non-thrombus group, and the difference was statistically significant (*P* < .05). Compared with the control group, the LASr, LAScd (Left Atrial Strain during Systolic Contraction), and LASct (Left Atrial Strain during Systolic Contraction and Twist) in the non-thrombus group and the thrombus group showed a decreasing trend.However, currently, there are few studies on the application of the 2D-STI technique to the function of the LAA. The LAA is a finger-like extension originating from the main body of the LA and can actively relax and contract.^[13]^ The compliance and blood storage capacity of the LAA are much higher than those of other parts of the LA.^[14]^ When AF occurs, the contractility of the LAA decreases, resulting in ineffective blood drainage and blood stasis. Meanwhile, the damage to the structure and function of the LA increases the chance of platelet aggregation. Moreover, the LAA has a fingerless blind-end structure with pectinate muscles distributed inside, making it a prone site for left atrial thrombus formation.^[15]^ Abnormal LAA function is also closely related to AF. Patients with AF have impaired mechanical function and reduced compliance in the LAA. Kupczynska et al^[16]^ conducted a retrospective study on 87 patients with AF and observed the LA longitudinal strain, systolic and early diastolic strain rates in the 4-chamber and 2-chamber views. Thirty-six patients (41%) underwent TEE examination, and it was found that the LAA strain obtained by 2D-STI could identify patients with a higher risk of LAA thrombus. Therefore, parameters obtained by TEE may provide auxiliary information for identifying LAA thrombus in patients with AF and may support clinical evaluation and treatment planning.^[17]^ Previous research results have shown^[18]^ that LAA deformation parameters can provide clinically useful information about LAA function before LAA structural remodeling occurs, and also provide additional diagnostic value for patients with LAA thrombus, with recognized sensitivity and specificity. In this study, while obtaining the left atrial strain parameters through 2D-STI, strain analysis of the LAA was also carried out using 2D-STI through TEE to obtain the global longitudinal strain of the LAA. Combined with the conventional 2-dimensional data of the LAA obtained by TEE, the functional structure of the LAA was evaluated. Moreover, previous studies on LAA strain applied the mode of left ventricular strain analysis. However, there are significant differences between the left ventricle and the LAA in terms of morphology and wall thickness. In this study, the LAAGLS (LAA Global Longitudinal Strain) was obtained through the RV Strain mode, and the right ventricle is closer to the LAA in both morphology and wall thickness. This study found that compared with the control group, there were statistically significant differences in LAAEV (LAA Emptying Velocity), LAAEF (LAA Emptying Fraction), and LAAGLS between the thrombus group and the non-thrombus group. This is consistent with the research results of Zuo et al^[19]^ confirming that the blood flow in the LAA of patients in the AF group is slow and the myocardial compliance is reduced, making them more prone to thrombus formation. Meanwhile, our study found that LAAEV had the strongest correlation with the CHA2DS2-VASc score, indicating that lower LAA emptying velocity was associated with the presence of LAA thrombus. LAAEV can be used as an evaluation indicator for the reservoir function and systolic function of the LA, including its contractility, stunning, and fibrosis. Moreover, LAAEV is also closely related to the contraction speed of the left atrial wall.

The ROC curve in this study showed that the areas under the curve of LASr, LAAEV, and LAD were 0.974, 0.793, and 0.714, respectively. The sensitivity of LASr was 72.2% and the specificity was 71.9%. The sensitivity of LAAEV was 86.1% and the specificity was 93.7%. The sensitivity of LAD was 91.7% and the specificity was 37.5%. These findings indicate that LAAEV is a useful discriminatory marker for distinguishing AF patients with and without LAA thrombus. Its Youden index was 0.798, and the cutoff value was 51.5 cm/s. In this study population, LAAEV <51.5 cm/s was associated with the presence of LAA thrombus. Our identified LAAEV cutoff of 51.5 cm/s is higher than several previously reported thresholds in the literature. Recent studies have reported varied LAAEV cutoff values for predicting LAA thrombus. Chen et al identified an LAAEV threshold of ≤21.5 cm/s with high diagnostic accuracy (area under the curve 0.88) in a cohort of 307 patients with non-valvular AF 2.^[20]^ Wang et al used <40 cm/s as a cutoff to define LAA dysfunction in their strain analysis study.^[21]^ Some earlier studies have employed even lower thresholds, with cutoff values around 20 cm/s being reported for detecting thromboembolic risk.^[22]^ The variation in reported LAAEV thresholds may be attributed to differences in measurement techniques, patient populations, and equipment specifications. Our higher threshold of 51.5 cm/s, while maintaining excellent diagnostic performance (sensitivity 86.1%, specificity 93.7%), may reflect the use of high-resolution TEE with advanced signal processing capabilities. Regarding comparative risk stratification models, recent studies have shown that the CHA2DS2-VASc score has limited discriminatory accuracy for identifying LAA thrombus. Segan et al reported that the CHA2DS2-VASc score had a predictive value of only 0.67 for LAA thrombus risk.^[23]^ Sun et al demonstrated that CHADS2 and CHA2DS2-VASc models had limited value in predicting LAA thrombus, with nomogram models showing superior performance.^[24]^ In our analysis, LAAEV achieved an area under the curve of 0.793, suggesting that this echocardiographic parameter has potential discriminatory value beyond conventional clinical risk stratification scores. While this threshold was derived from TEE examinations that also visualized thrombus, the quantitative LAAEV measurement provides standardized, objective assessment of LAA function that can complement visual thrombus detection. This approach may be particularly valuable for: standardizing LAA function assessment across different operators and institutions, identifying patients with severe LAA dysfunction who warrant more frequent monitoring even in the absence of visible thrombus, and providing guidance for clinical decision-making when TEE is not immediately available or technically challenging to perform.

Beyond structural and mechanical remodeling, AF is sustained by interacting molecular and electrophysiological mechanisms. Epigenetic regulation, including changes in microRNA expression, has been implicated in atrial electrical and structural remodeling and may also be modified after catheter ablation.^[25]^ Inflammation and oxidative stress represent additional components of the AF substrate and may contribute to atrial remodeling and recurrence after ablation. In a randomized study of patients undergoing catheter ablation, α-lipoic acid reduced circulating inflammatory and oxidative stress markers, including C-reactive protein and tumor necrosis factor-α, although this biological effect did not translate into a significant reduction in AF recurrence, highlighting the multifactorial nature of post-ablation recurrence.^[26]^ Abnormal intracellular calcium handling may represent another relevant mechanism. In patients with persistent AF undergoing epicardial thoracoscopic ablation, the CAMAF study reported an association between sarcoplasmic/endoplasmic reticulum Ca^2+^-ATPase (SERCA) levels and ablation response, suggesting that impaired calcium homeostasis may contribute to AF persistence and resistance to rhythm-control interventions.^[27]^

Autonomic and cardiometabolic abnormalities may further modulate this arrhythmogenic substrate, particularly in high-risk populations. In patients with type 2 diabetes, cardiac autonomic dysfunction has been associated with otherwise asymptomatic or brief AF episodes, supporting a potential contribution of sympathovagal imbalance to AF susceptibility in metabolically vulnerable patients.^[28]^ More recently, a prospective study of patients with type 2 diabetes undergoing AF ablation reported that combined treatment with sodium–glucose cotransporter-2 inhibitors and glucagon-like peptide-1 receptor agonists was associated with lower AF recurrence and reduced inflammatory and oxidative stress markers compared with either therapy alone.^[29]^ Collectively, these observations indicate that atrial dysfunction in AF reflects the integrated effects of structural remodeling, inflammation, altered calcium handling, autonomic imbalance, and cardiometabolic stress. In the present study, these pathways were not directly measured; therefore, the reductions in LA and LAA functional parameters observed in patients with AF, particularly those with LAA thrombus, should be considered integrated phenotypic manifestations of atrial remodeling rather than evidence of any specific molecular mechanism.

Regarding the study design, this study should be interpreted as a comparative cross-sectional observational study. The AF patients were consecutively screened from a clinical population scheduled for radiofrequency ablation, and thrombus status and echocardiographic parameters were assessed during the same preoperative evaluation period. The thrombus and non-thrombus groups were formed after transesophageal echocardiographic assessment rather than through intentional case-control recruitment. The healthy reference group was included only for comparison of echocardiographic parameters and was not used in the regression analysis for LAA thrombus.

### 4.1. Clinical applications and implications

The high sensitivity (86.1%) and specificity (93.7%) of LAAEV at the optimal cutoff of 51.5 cm/s have significant clinical implications for managing patients with non-valvular AF. The sensitivity of LAAEV suggests that this parameter may provide auxiliary information when evaluating the likelihood of LAA thrombus in patients with non-valvular AF. This could lead to cost savings and reduced patient discomfort while maintaining diagnostic accuracy. The specificity of LAAEV indicates that patients with LAAEV values ≤51.5 cm/s were more likely to have LAA thrombus in this study population, suggesting the need for careful TEE assessment and individualized anticoagulation evaluation before interventional procedures such as radiofrequency ablation. The Youden index of 0.798 suggests that LAAEV may be considered as an auxiliary echocardiographic marker to support clinical assessment, although its application in clinical decision algorithms requires further prospective validation. Furthermore, the noninvasive nature of 2D-STI supports the feasibility of incorporating related functional parameters into routine clinical evaluation, but their impact on patient outcomes requires further validation.

### 4.2. Limitations of this study

In the 2D-STI analysis, there is no specialized software for the LAA. In this study, the right ventricular speckle tracking software was used for the analysis. Although the morphology of the right ventricle is closer to that of the LAA than that of the left ventricle and its wall thickness is also closer to that of the LA, the morphology of the LAA varies greatly, so there may be differences between the obtained results and the actual situation.

The sample size of AF patients was relatively small (n = 68), which limited the number of variables that could be reliably included in the multivariable logistic regression model. Although we adjusted for clinically relevant covariates, including CHA_2_DS_2_-VASc score, LAD, and LVEF, the regression results may still be affected by model instability or overfitting and should therefore be interpreted with caution. In addition, this study lacked long-term follow-up, and future larger prospective studies are needed to validate these findings and to observe whether thromboembolic events occur in patients with LAA thrombus.

Potential circularity in study design: Our study measured LAAEV and identified thrombus using the same TEE examination, which may limit the predictive value of our findings. Future prospective studies should validate these thresholds in clinical scenarios where LAAEV measurement precedes thrombus assessment, such as in patients undergoing serial monitoring or when TEE is not immediately available.

Clinical application requires clarification: While our study demonstrates the relationship between LAAEV and thrombus presence, the clinical utility of this approach needs further validation in scenarios where it provides incremental value beyond direct TEE visualization of thrombus.

The cutoff value for LAAEV was derived and evaluated from the same dataset, which may introduce optimism bias. External validation in an independent cohort would be needed to confirm the generalizability of our findings.

Because this was a single-center comparative cross-sectional study, the temporal relationship between echocardiographic parameters and LAA thrombus formation could not be determined. Therefore, the findings should be interpreted as associations rather than causal relationships, and further prospective cohort studies are needed to validate the predictive value of these parameters.

Because this study defined the thrombus group according to the presence of LAA thrombus, potential misclassification could not be completely excluded if thrombus was present in the LA body but not in the LAA. Such misclassification may dilute the observed associations between echocardiographic parameters and LAA thrombus status. Future studies should separately record and analyze thrombi located in the LA body and LAA to further clarify the clinical significance of LA and LAA functional parameters.

## 5. Conclusion

In conclusion, the combination of 2D-STE and TEE can help clinicians comprehensively evaluate LA and LAA functional changes in patients with AF. In this study, LAAEV and LASr were the main echocardiographic markers associated with the presence of LAA thrombus. These findings may provide useful reference information for clinical assessment and management of patients with non-valvular AF, although further prospective studies are needed to confirm their clinical utility.

## Acknowledgments

We sincerely thank all staff and patients who participated in this research, as well as all individuals and institutions that provided support.

## Author contributions

**Data curation:** Qiankun Chang.

**Formal analysis:** Qiankun Chang.

**Investigation:** Limin Lu.

**Methodology:** Limin Lu.

**Software:** Limin Lu.

**Resources:** Wenying Wu.

**Project administration:** Liang Fang.

**Supervision:** Liang Fang, Peiming Feng.

**Validation:** Chunqiang Bai.

**Visualization:** Chunqiang Bai.

**Conceptualization:** Peiming Feng.

**Writing – original draft:** Xianghong Guan.

**Writing – review & editing:** Xianghong Guan, Wenying Wu.
